# Interlaboratory evaluation of plasma *N*-glycan antennary fucosylation as a clinical biomarker for HNF1A-MODY using liquid chromatography methods

**DOI:** 10.1007/s10719-021-09992-w

**Published:** 2021-03-25

**Authors:** Daniel Demus, Bas C. Jansen, Richard A. Gardner, Paulina A. Urbanowicz, Haiyang Wu, Tamara Štambuk, Agata Juszczak, Edita Pape Medvidović, Nathalie Juge, Olga Gornik, Katharine R. Owen, Daniel I. R. Spencer

**Affiliations:** 1 Ludger Ltd, Culham Science Centre, Oxfordshire Abingdon, UK; 2 Center for Proteomics and Metabolomics, Leiden University Medical Center, Leiden, The Netherlands; 3 Quadram Institute Bioscience, Norwich Research Park, Norwich, UK; 4 Genos Glycoscience Research Laboratory, Zagreb, Croatia; 5 Faculty of Pharmacy and Biochemistry, University of Zagreb, Zagreb, Croatia; 6 Oxford Centre for Diabetes, Endocrinology and Metabolism, University of Oxford, Oxford, Oxfordshire UK; 7 Vuk Vrhovac University Clinic for Diabetes, Endocrinology and Metabolic Diseases, Merkur University Hospital, Zagreb University School of Medicine, Croatia School of Medicine, Zagreb, Croatia; 8 Oxford NIHR Biomedical Research Centre, Oxford Hospitals NHS Foundation Trust, Oxford, Oxfordshire UK

**Keywords:** HNF1A-MODY, Glycosylation, Biomarker, Diagnostics, N-glycans, Fucosylation

## Abstract

Antennary fucosylation alterations in plasma glycoproteins have been previously proposed and tested as a biomarker for differentiation of maturity onset diabetes of the young (MODY) patients carrying a functional mutation in the *HNF1A* gene. Here, we developed a novel LC-based workflow to analyze blood plasma N-glycan fucosylation in 320 diabetes cases with clinical features matching those at risk of HNF1A-MODY. Fucosylation levels measured in two independent research centers by using similar LC-based methods were correlated to evaluate the interlaboratory performance of the biomarker. The interlaboratory study showed good correlation between fucosylation levels measured for the 320 cases in the two centers with the correlation coefficient (r) of up to 0.88 for a single trait A3FG3S2. The improved chromatographic separation allowed the identification of six single glycan traits and a derived antennary fucosylation trait that were able to differentiate individuals carrying pathogenic mutations from benign or no *HNF1A* mutation cases, as determined by the area under the curve (AUC) of up to 0.94. The excellent (r = 0.88) interlaboratory performance of the glycan biomarker for HNF1A-MODY further supports the development of a clinically relevant diagnostic test measuring antennary fucosylation levels.

**Supplementary Information:**

The online version contains supplementary material available at 10.1007/s10719-021-09992-w.

## Introduction

Glycosylation is a co-/post-translational modification which affects protein conformation and interactions. Glycans can be covalently attached to proteins through either N- or O-glycosidic linkages. These oligosaccharide chains strongly influence protein-protein interactions and are involved in protein folding, sub-cellular targeting, and trafficking. N-glycans are linked to the amide group of asparagine (Asn), whereas O-glycans are attached to the hydroxyl group of serine (Ser) or threonine (Thr) in the polypeptide backbone [1].

Aberrant protein glycosylation has been associated with many pathological conditions in humans. Some chronic inflammatory [2], autoimmune [3] and infectious diseases [4], such as inflammatory bowel disease [5], rheumatoid arthritis [6] and HIV infection [7] were associated with changes in N- and/or O-glycosylation profiles of plasma proteins. Glycan signatures have been extensively studied in the field of cancer research, with altered sialylation [8], galactosylation [9] and fucosylation [10] identified in various cancer types. These studies showed the potential of using glycans as biomarkers to screen for certain pathological conditions, monitor patients undergoing treatment or predict occurrence or reoccurrence of a disease even before any physiological symptoms can be detected [11–16].

HNF1A-MODY (MODY3) is a type of maturity onset diabetes of the young (MODY) for which a glycan biomarker has been proposed for patient identification and stratification [17, 18]. HNF1A-MODY is the most common form of autosomal dominant monogenic diabetes in adults, accounting for around 50 % of all MODY cases in the UK [19]. It is caused by mutations in the *HNF1A* gene which encodes for the HNF-1α protein, a transcription factor regulating expression of several genes that are important for pancreatic β-cell development and function [20]. Different rare variants (classified as damaging, likely-damaging and benign) in the *HNF1A* gene cause various severity of the disease. Damaging (loss-of-function, pathogenic) mutations in the *HNF1A* cause deregulation of β-cell genes and these mutations have been shown to result in generation of abnormal β-like cells *in vitro* [21]. The disease is characterized by progressive deterioration of β-cell function, but insulin sensitivity remains normal or slightly increased [22, 23]. Clinically, individuals with HNF1A-MODY are at increased risk of vascular complications due to hyperglycaemia [24].

Although glycomics studies have demonstrated the ability to systematically differentiate HNF1A-MODY patients from other diabetes forms [17, 18], there has not yet been widespread adoption of this approach in clinical environments and currently, testing for MODY depends on the awareness and interest of individual clinicians. A MODY probability calculator based on clinical features has been developed to help clinicians identify those with the highest risk of MODY [25]. The presence of *HNF1A* mutations requires genetic testing but the diverse range of mutations is not completely predictive of changes in the protein function [26, 27]. In addition, due to the cost and unavailability of genetic testing in many countries, many patients remain misdiagnosed or unidentified. Importantly, misdiagnosis often results in patients being referred to an inappropriate course of treatment [28].

Genome-wide association studies (GWAS) have shown that C-reactive protein (CRP) expression and N-glycan antennary fucosylation levels are significantly altered in patients harbouring mutations in the *HNF1A* gene [29]. Changes in antennary fucosylation are a result of altered activity of specific fucosyltransferases catalyzing the α-1,3 or α-1,4 addition of fucose in the antennary N-acetylglucosamines of N-glycans. Previous studies that have determined the levels of N-glycan fucosylation in blood plasma using liquid chromatography (LC) methods showed the excellent performance of the glycan biomarker in differentiating HNF1A-MODY patients in large patient cohorts [17, 18].

Due to the evolution of liquid separation techniques since the first HNF1A-MODY study [17], it is now possible to perform a more exhaustive evaluation of potential glycan biomarkers. Furthermore, N-glycan antennary fucosylation as a biomarker for HNF1A-MODY has not been evaluated in the context of its robustness and repeatability of measurements between laboratories. Assessment of interlaboratory performance of the biomarker will provide further valuable insights into its clinical application.

Here, we evaluated the interlaboratory performance of plasma protein antennary fucosylation as a biomarker for HNF1A-MODY based on a set of 320 patients with non-autoimmune diabetes from the cohort previously analyzed [18]. We analyzed glycans present in the blood plasma by using a newly developed workflow employing a LC method with fluorescence (FD) and mass spectrometric (MS/MS) detection (LC-FD-MS/MS) supported by exoglycosidase digestions. Subsequently, we compared fucosylation levels for 320 individuals that were measured in the current study and analyzed previously as a part of the study by Juszczak *et al*. [18] using LC-based methods in two research centers. In addition to the individual N-glycan structures, a derived antennary fucosylation trait was calculated and tested for its diagnostic potential.

## Materials and methods

### Sample cohort

The research presented here was performed using blood plasma samples obtained from study participants that were recruited via the Young Diabetes in Oxford (YDX) study in the UK (n = 90) and the Croatian National Diabetes Registry (CroDiab) in Croatia (n = 230). Subjects older than 18 years who were diagnosed with diabetes before age of 45 years were eligible to take part in the study. Biochemical inclusion criteria were fasting C-peptide ≥ 0.2 nmol/L, which indicates insulin production, and negative GAD antibodies (GADA: the commonest antibody found in type 1 diabetes). Informed consent was obtained for all participants. Clinical characteristics of patients included in the study are summarized in Table 1.

**Table 1:** Clinical characteristics of patients included in the study

	(Likely) damaging allele n = 18 cases	VUS n = 5 cases	(Likely) benign n = 8 cases	No rare *HNF1A* allele variant n = 289 cases
Sex, male:female [%]	28:72	40:60	50:50	52:48
Age at recruitment [years]	39.7 (17.4)	54.2 (14.2)	48.1 (11.2)	45.6 (9.7)
Age at diagnosis [years]	25.7 (8.6)	37.3 (9.8)	36.4 (5.6)	35.3 (6.5)
Diabetes duration [years]	13.1 (11.6)	17.3 (10.6)	9.1 (6.6)	10.3 (7.7)
BMI [kg/m2]	25.93 (4.75)	28.34 (6.19)	34.61 (5.02)	29.95 (5.76)
hsCRP [mg/L]	0.80 (1.32)	4.01 (6.30)	5.21 (5.75)	3.79 (6.97)
HbA1c [%]	7.7 (1.7)	6.7 (1.3)	8.8 (2.1)	7.6 (1.8)
C-peptide [nmol/L]	0.42 (0.21)	0.60 (0.57)	0.81 (0.62)	0.75 (0.36)
Total cholesterol [mmol/L]	4.75 (1.09)	4.74 (1.21)	5.07 (0.88)	4.79 (1.36)
HDL [mmol/L]	1.37 (0.32)	1.28 (0.39)	1.11 (0.23)	1.17 (0.36)
Triglycerides [mmol/L]	1.24 (0.51)	1.12 (0.31)	1.98 (0.96)	2.15 (1.98)

Mean values are presented for each group (standards deviation (SD) in parentheses)

Plasma samples were obtained from patients’ blood. All cases underwent Sanger sequencing for HNF1A-MODY as part of the MODY-glycan study [18]. Plasma samples were grouped based on *HNF1A* mutation type, which was allocated using a systematic and functional assessment of rare *HNF1A* alleles. These included three mutation groups: (likely) damaging, variants of unknown significance (VUS), (likely) benign and a group of patients without HNF1A mutation (D).

### *N*-glycan release, fluorescent labelling and purification

Enzymatic release and fluorescent labelling of N-glycans from human blood plasma samples were performed using a highly automated analytical workflow for high-throughput (HTP) glycomics supported by a Hamilton STARlet liquid handling robot, as described previously [30, 31] with minor modifications. Here, we used 4 µL of human blood plasma samples and a procainamide labelling kit with sodium cyanoborohydride as the reductant (LT-KPROC-96, Ludger) for fluorescent labelling of released N-glycans.

### Exoglycosidase digestion

Procainamide-labelled plasma N-glycan samples (8 µL) were treated with either 1 µM E1_10125 α-L-fucosidase from Ruminoccocus gnavus strain E1 (E1_10125) with specificity towards α1–3,4 > 2 fucose [32] or 1 µL bovine kidney α-L-fucosidase (BKF; Sigma; specific towards α1–6 > 2,3,4 fucose) [33]. The enzymatic reaction was carried out in sodium phosphate buffer (50 mM, pH 6.0) at 37 °C for 18 h. Digested N-glycans were purified using LT-KPROC-96 plate (Ludger), eluted in 200 µL of water, evaporated to dryness and reconstituted in 100 µL of water. During the procedure, an enzymatically untreated aliquot of procainamide-labelled plasma N-glycans was used as a process control.

### HILIC-LC-FD-MS/MS analysis of PROC-labelled glycans

Procainamide-labelled N-glycan samples were analyzed by LC-FD-MS/MS with electrospray ionization (ESI). Prior to analysis, samples were freshly prepared in a 96-deep well collection plate (LP-COLLPLATE-2ML-96; Ludger) on the Hamilton STARlet liquid handling robot as a mixture of water and acetonitrile in a 1:3 ratio. To verify optimal LC-MS signals, a procainamide-labelled plasma N-glycan standard from a readily available source was used as a system suitability standard prior to each analysis. The readily available standard had been prepared from pooled human plasma (Sigma Aldrich) according to a standard protocol (described in N-glycan release, fluorescent labelling and purification), aliquoted, stored at -21 °C before use.

Each sample (25 µL) was injected onto a HALO 2 Penta-HILIC 150 × 2.1 mm column with 2.0 μm stationary phase particle size (AMT91812-705, Advanced Materials Technology) at 40 °C on a Dionex Ultimate 3000 UHPLC instrument with a fluorescence detector (excitation and emission wavelengths of 310 and 370 nm, respectively), coupled in-line to a Bruker Amazon Speed ETD mass spectrometer. Data acquisition was controlled by HyStar version 3.2 (Bruker). Solvent A was 200 mM ammonium formate buffer (pH 4.4) (LS-N-BUFFX40, Ludger), solvent B was acetonitrile and solvent C was pure water.

A 70-min run was used with a multi-step gradient consisting of 7–44 % solvent A, 72 − 56 % solvent B, 21 − 0 % solvent C over 62 min at a flow rate of 0.4 ml/min, followed by 44–50 % A, 56 − 0 % B, 0–50 % C in 3.5 min at a flow rate of 0.3 mL/min, returning to 7 % A, 72 % B and 21 % C over 4 min at a flow rate of 0.45 mL/min. The mass spectrometer was used in enhanced resolution mode, positive ion setting, at range of 400–1700 m/z. Other settings were as follows: nebulizer pressure 14.5 psi, capillary voltage 4500 V, nitrogen flow 10 L/min, ion charge control (ICC) target 200,000, maximum accumulation time 50.00 ms, singly charged ions excluded.

### Data processing and statistical analysis

Bruker Compass DataAnalysis version 4.1 and Bruker ProteinScape version 4.0 software (GlycomeDB database) were used to analyze mass spectrometry (MS) data. Glycan structures were identified based on combination of LC-FD-MS/MS data supported by exoglycosidase digestion data, the UHPLC column separation characteristics and data from the literature [34]. GlycoWorkBench version 2 was used for searching most plausible glycan structures based on accurate mass. Fluorescence traces were exported as an open text format using Chromeleon version 7.2 (Thermo Fisher). UHPLC data processing and quantification was performed using HappyTools version 0.0.2 build 180521a [35]. A feature list, containing peak retention times and widths, was generated using the automated peak detection option of HappyTools. Subsequently, the feature list was manually curated after visual inspection of the overlaid chromatograms. The main features were selected and used for calibration of all the chromatograms, while the entire feature list was used for HTP quantification of all the detectable features (minimum intensity = 0.001; Sigma edge method; Sigma value = 2.0) in the chromatograms. Analyte and chromatogram QC parameters were also determined using HappyTools. These parameters were then used to support manual curation of the results, evaluating the Gaussian peak quality (GPQ ≥ 0.8) and signal-to-noise (SN ≥ 9) ratio for each LC feature.

Statistical analysis and visualization were performed using Microsoft Excel and R platform version 1.1.463. Bonferroni correction was applied to test for sex and age effect. All tests were significant after Bonferroni correction (number of tests n = 183; 6 potential markers with sex, age and, sex*age correction; p cut-off = 2.78 × 10-2). For Receiver Operating Characteristic (ROC) curve analysis, (likely) damaging, (likely) benign and no *HNF1A* mutation groups were tested against each other. Single glycan traits were tested for the best classification performance. ROC curves, area under the curve (AUC), optimal sensitivity, specificity and cutoff values were generated using "cutpointr" R package. A derived trait, which averages antennary fucosylation feature across individual glycan structures, was tested separately. Data outliers were identified with the 1.5xIQR rule [36]. Outliers were detected for each glycan trait per patient group before ROC analysis.

Data collected as a part of the previous study by Juszczak *et al*. [18] using the same sample cohort was used to evaluate the interlaboratory performance of the glycan biomarker by Spearman correlation method. In the previous study, the relative abundance of glycans in each glycan peak was expressed as a percentage of the total integrated area. Here, fucosylation levels were expressed in fucosylation indexes that were calculated for each glycan trait (Table 2). The best performing glycan traits, corresponding to the same glycan structure (GP30 – A3FG3S2, GP36 – A3FG3S3, GP38 – FA3FG3S3) in both studies, were subjected to the correlation analysis.

**Table 2:**
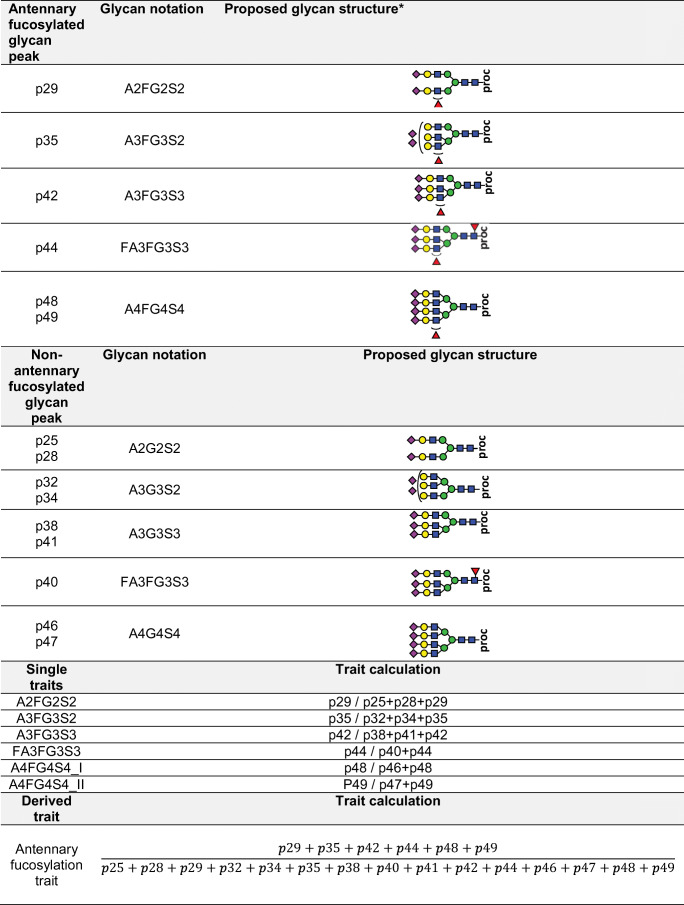
Construction of direct and derived antennary fucosylation traits

*the graphical representations of glycan structures used the Symbol Nomenclature for Glycans (SNFG): blue square (N-acetylglucosamine), green circle (mannose), yellow circle (galactose), purple diamond (N-acetylneuraminic acid), red triangle (fucose) [37, 38]

## Results

### Development and validation of a novel workflow for identification and characterization of antennary fucosylation of human plasma proteins

To determine the antennary fucosylation levels of 320 subjects with diabetes, we developed a workflow encompassing highly reproducible ultra-high-performance liquid chromatography (UHPLC) analysis and high-throughput (HTP) systematic data processing. This approach allows a highly robotized, simultaneous analysis of 96 samples made up of 93 clinical samples and a pooled human plasma standard in triplicate as a control.

Our LC-FD-ESI-MS/MS-based approach included the use of a HILIC-type UHPLC column that separates glycans depending on their size and degree of sialylation coupled with exoglycosidase digestions for glycan structure identification (Supplementary Figure S1). The use of a column packed with core–shell silica particles, which provides lower backpressure, was implemented to achieve a desired glycan separation without excessively long run times [39]. Definitive identification of fucosylated N-glycan structures was achieved using a combination of FD-MS/MS detection and exoglycosidase digestions. Analysis of the fragmentation mass spectra (MS) was performed with a focus on the following ions: m/z 512.19 for the GlcNAc-Gal-Fuc fragment and m/z 587.33 for the PROC-GlcNAc-Fuc fragment, which indicate antennary and core fucosylation of N-glycans, respectively. Since fucose migration may lead to misleading readouts of MS/MS data [40, 41], exoglycosidase digestions were used to detect and distinguish the presence of antennary and core fucosylation. The penta-HILIC column provided separation of various glycan isomers, specifically sialic acid linkage isomers, revealing two tetra-sialylated antennary-fucosylated structures (A4FG4S4). We conclude that the two A4FG4S4 glycan structures are sialic acid linkage isomers, however determination of sialic acid linkages (α-2,3 or α-2,6) is not possible as no sialic acid derivatization approach was applied in this study. Forty-nine glycan peaks were assigned (Supplementary Figure S2 and Supplementary Table S1) and 6 peaks were identified as antennary fucosylated structures: p29, p35, p42, p44, p48, p49, corresponding to A2FG2S2, A3FG3S2, A3FG3S3, FA3FG3S3 and two isomers of A4FG4S4 respectively.

Antennary fucosylated glycan peaks and their non-fucosylated counterpart peaks were used for calculations of traits that were subsequently applied to measure fucosylation levels in blood plasma samples. The glycan peaks used for analyses and calculated traits are listed and summarized in Table 2. Glycosylation traits were calculated based on relative peak areas normalized to the peaks used for statistical analysis (Supplementary Table S2).

To evaluate technical variations within the analytical approach, pooled human plasma was used as an internal control in triplicate in order to monitor consistency of sample preparation and data processing steps. The triplicates were also used to evaluate repeatability of the glycan analysis (Supplementary Table S2). The most abundant peak was p28 with a coefficient of variation (CV) of 1 %. Peaks with relative intensities (RI) above 1 % (p25, p28, p29, p32, p34, p38, p41, p42, p46 and p47) gave the average CVs of 7 % and 5 % for the inter- and intra-plate studies, respectively.

### Interlaboratory evaluation of the glycan biomarker for classification of HNF1A-MODY

The workflow developed and described above was applied to plasma samples collected as part of a previous cohort from patients in the UK and Croatia, described elsewhere [18]. Based on the presence and assessed disease-causality of *HNF1A* mutations, participants were divided in four groups: (likely) damaging (n = 18), (likely) benign (n = 8) and VUS (n = 5), and a group of no *HNF1A* mutation diabetes cases (n = 289). Antennary fucosylated glycan levels were measured and subsequently correlated for the same set of cases (n = 320) using similar LC-based workflows in two independent laboratories, one in the UK and one in Croatia.

**Fig. 1:**
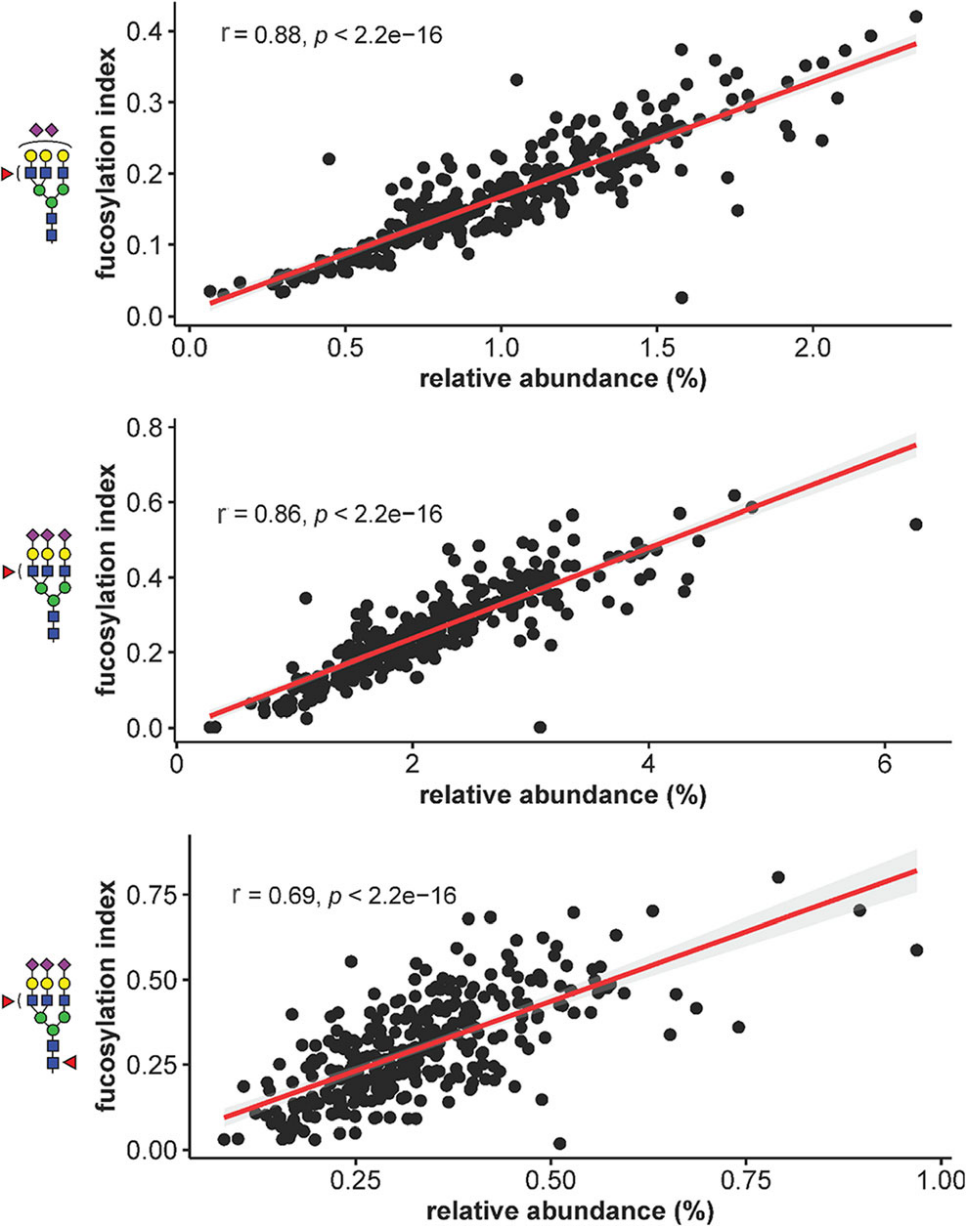
Correlation analysis illustrating the performance of three single glycan traits used as differentiating biomarkers for HNF1A-MODY in two independent laboratories. Antennary fucosylation levels measured as the relative abundance of antennary fucosylated glycans [18] or as fucosylation indexes (current study) were compared for 320 individuals with diabetes. The performance of each glycan trait is described by the Spearman’s correlation coefficient (r)

We compared three directly measured, antennary fucosylated glycans – A3FG3S2 (chromatographic peak GP30 in Juszczak *et al*., p35 herein), A3FG3S3 (GP36, p42) and FA3FG3S3 (GP38, p44), which showed the best discriminative performance for HNF1A-MODY in the study by Juszczak *et al*. [18]. The analysis showed a significant correlation for the antennary fucosylated glycan levels expressed as either the relative abundance (percentage of a certain N-glycan structure in the total plasma N-glycome) [18] or as antennary fucosylation indexes (current study). The Spearman’s correlation coefficient (r) ranged between 0.69 and 0.88 (Fig. 1). The best results were obtained for antennary fucosylated structures A3FG3S2 and A3FG3S3 with r scores of 0.88 and 0.86, respectively. The core and antennary fucosylated glycan structure (FA3FG3S3) trait gave the r score of 0.69.

### Identification of novel glycosylation traits as biomarkers of HNF1A-MODY

Glycosylation trait analysis was carried out to identify single fucosylated glycan structures that could discriminate between a pathogenic group of (likely) damaging (n = 18) mutation cases and nonpathogenic groups of either benign (n = 8) or no *HNF1A* mutation (n = 289) cases.

**Fig. 2:**
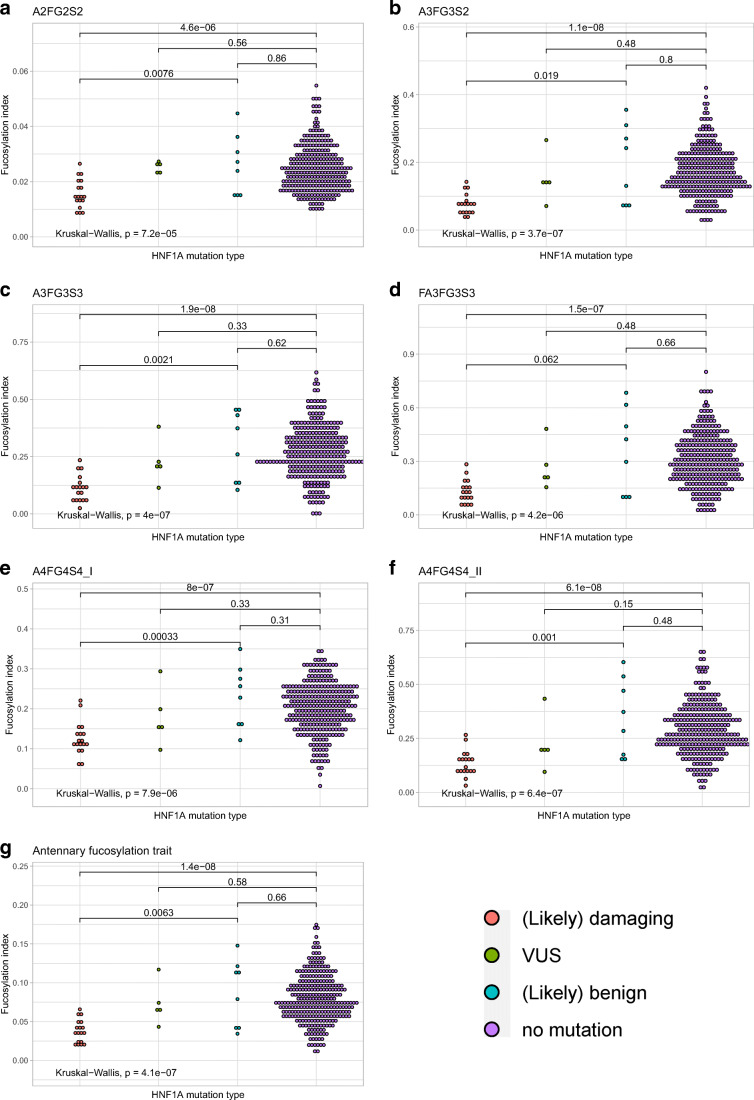
Dot plots presenting antennary fucosylation levels measured using each glycosylation trait for groups of individuals without *HNF1A* mutation and with different *HNF1A* mutation types. The lines and numbers above the box plots, indicate the p-value when comparing two categories using The Wilcoxon-Mann-Whitney test. The analysis with p ≤ 0.05 is considered statistically significant

We evaluated differences in antennary fucosylation levels between *HNF1A* mutation groups measured by applying single traits (A3FG3S2, A3FG3S3, FA3FG3S3, A4F4G4_I, A4G4S4_II) and the derived trait (Fig. 2). A3FG3S2, A3FG3S3, FA3FG3S3, A4F4G4_I, A4G4S4_II and the derived antennary fucosylation trait allow for differentiation of the pathogenic mutation group from the nonpathogenic mutation groups, by showing significant differences (p ≤ 0.05) in fucosylation levels between these groups. Individuals with (likely) damaging mutations in the HNF1A gene present significantly decreased antennary fucosylation levels in respect to benign and no mutation groups. No significant difference was observed between (likely) benign and no *HNF1A* mutation groups for all traits.

**Fig. 3:**
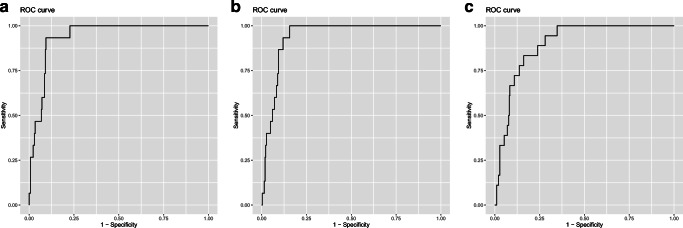
ROC curves illustrating the performance of single glycan traits (**a** and **b**) and the derived antennary fucosylation trait (**c**) in differentiating patients with (likely) damaging *HNF1A* mutation from a group of patients without *HNF1A* mutation. The AUC values are displayed for the best performing single glycan traits and the derived trait

Receiver Operating Characteristic (ROC) curves were used to assess the diagnostic accuracy of glycan traits for discrimination of *HNF1A* mutation types. As a result of data curation, out of the 320 samples tested, 24 samples (including 3 pathogenic cases) for single traits and 4 samples for the derived trait were classified as outliers prior to ROC analysis. ROC analysis showed that all tested traits provided good discriminative power between pathogenic and no *HNF1A* mutation cases, as determined by the measure of area under the curve (AUC) found to be in a range of 0.84 to 0.94 (S3 Table). A3FG3S2 and A3FG3S3 performed best within the single traits providing AUCs of 0.94 with 93 % sensitivity, 87 % specificity at the cutoff of 0.101 and 100 % sensitivity and 83 % specificity at the cutoff of 0.163, respectively (Fig. 3a − 3b). The derived trait performed well in differentiating pathogenic cases from no *HNF1A* mutation group with an AUC of 0.90, 83 % sensitivity and 78 % specificity at the cutoff of 0.057 (Fig. 3c). By applying these three markers, one case within the VUS group (n = 5) would have been classified as pathogenic. Two novel single traits based on isomers of A4FG4S4 glycan gave AUCs of 0.90 and 0.93 for A4FG4S4_I and A4FG4S4_II, respectively, when distinguishing between pathogenic and no *HNF1A* mutation groups. When testing (likely) damaging cases against (likely) benign group, the glycan traits gave AUCs in a range of 0.76 to 0.96 with A4FG4S4_I, providing the best discriminative power within the single traits (S3 Table).

More than half of all antennary fucosylated N-glycans in human blood plasma derive from α-1-acid glycoprotein (AAG), an acute phase protein [42, 43]. Correlation between CRP, AAG, and other inflammatory proteins has been previously reported [44], therefore CRP levels were checked within (likely) damaging *HNF1A* (n = 15) and no *HNF1A* mutation (n = 267) cases included in the study, and (likely) damaging *HNF1A* (n = 3) and no *HNF1A* mutation (n = 21) cases classified as data outliers, excluded from the ROC analysis. Increased CRP levels, regarding the mean value calculated within (likely) damaging cases included in the ROC analysis, were observed for the outliers, however, the difference was not considered statistically significant (S4 Table). Furthermore, increased rates of α1,3-fucosylation of tri-antennary and tetra-antennary glycans detected in sera of patients with chronic inflammation have been reported [45]. In support of these findings we observed significant positive correlations for the glycan traits based on tri- and tetra-antennary structures (A3FG3S2, A3FG3S3, FA3FG3S3, A4FG4S4_I, A4FG4S4_II) as well as the derived fucosylation trait and CRP levels within the cohort (S5 Table).

## Discussion

Individuals with HNF1A-MODY carry variants in the *HNF1A* gene, encoding for a transcription factor HNF1α, which has been found to be a master regulator of plasma protein fucosylation [29]. Expression of genes encoding for enzymes involved in L-fucose biosynthesis and several fucosyltransferases (FUTs) is under the regulation of HNF1α. In the presence of loss-of-function variants in the *HNF1A* gene, repressing activity of HNF1α towards FUT8 decreases. This leads to upregulation of core fucosylation in an α-1,6 linkage to the first GlcNAc residue in N-glycans and downregulation of antennary fucosylation in α-1,3 and α-1,4 linkage positions catalyzed by FUT3, FUT5 and FUT6 [46]. Based on these findings, the decrease in α-1,3 and α-1,4 antennary fucosylation of N-glycans from blood plasma protein has been proposed as a biomarker to identify individuals with HNF1A-MODY [17].

In this study, we analyzed N-glycan fucosylation of plasma glycoproteins in 320 individuals with diabetes and clinical features matching those at risk of HNF1A-MODY, using a newly developed LC-based workflow. The new workflow provides an excellent chromatographic separation of antennary fucosylated structures and is enhanced by the use of novel exoglycosidases. Unlike other commercially available antennary fucosidases, the novel E1_10125 enzyme is characterized by activity towards α-1,3/4 linkage fucosylated substrates presenting a terminal sialic acid modification [32]. By applying E1_10125 and the commercially available α-1,6 linkage specific fucosidase (BKF, Sigma), we were able to fully distinguish between antennary and core fucosylation of N-glycan structures. Retaining terminal sialic acid residues enabled the analytical method used to maintain the improved chromatographic separation power and variations in specific isoforms. Using this analytical approach, most antennary fucosylated structures found on human blood plasma proteins [34] were tested for their clinical potential as HNF1A-MODY biomarkers.

The interlaboratory performance of the glycan-based biomarker for HNF1A-MODY was determined by correlating antennary fucosylation levels measured using single glycan traits for the same set of samples by using similar LC methods in two independent research centers. Different UHPLC columns, sample processing and glycan separation conditions were applied in both centers. 2-aminobenzamide-labelled and procainamide-labelled glycans were analyzed by Juszczak *et al*. and in the current study, respectively [18]. Despite technical and methodological differences in the analytical workflows, the evaluation study proved the robustness and reproducibility of antennary fucosylated N-glycans as biomarkers for HNF1A-MODY, as shown by a strong correlation for the three glycan traits. The traits based on antennary fucosylated glycan structures (A3FG3S2 and A3FG3S3) gave very consistent readouts of fucosylation levels within 320 samples for the two laboratories with the correlation coefficient (r) ranging from 0.86 to 0.88. The trait based on the glycan structure containing both core and antennary fucosylation (FA3FG3S3) performed the worst in the interlaboratory study. Previously, the opposite effect that mutations in the *HNF1A* gene have on core and antennary fucosylation has been reported [29]. Therefore, taking into account the methodological differences such as trait calculation in both studies, we conclude that core fucosylation and the presence of an additional antennary fucosylated glycan structure (A4FG4S3) under the peak GP38 might have had an impact on the worser performance of FA3FG3S3.

Furthermore, the new analytical workflow allowed us to identify six single antennary fucosylated glycan traits that were tested for their clinical potential to discriminate between patients with pathogenic and nonpathogenic mutations in the *HNF1A* gene, or without them. The best performing single glycan traits were found to be A3FG3S2 and A3FG3S3, both giving an AUC of 0.94, for classifying pathogenic mutation cases against a group of cases without the *HNF1A* mutation. The same single glycan traits were also the best performing ones in the study by Juszczak *et al*. [18], achieving an AUC of 0.90 for both A3FG3S2 and A3F2G3S3. This minor difference in classification performance assessed by ROC curve analysis is due to different study design, since the previous study considered a larger group of subjects with non-autoimmune diabetes and no *HNF1A* mutations (n = 960), while herein only a subset of the group was analyzed (n = 289).

Thanabalasingham *et al*. previously proposed a derived trait (Dg-9 index), obtained by averaging antennary fucosylated triantennary glycans against all tri-antennary glycans, which was evaluated for the discrimination of HNF1A-MODY from groups of well-defined patients with a diagnosis based on molecular, biochemical and clinical investigation [17]. Here, the improved chromatographic separation allowed us to calculate and subsequently test the derived trait, which reflects overall changes in antennary fucosylation levels of blood plasma proteins better than a single trait. The derived antennary fucosylation trait performed well in differentiating pathogenic *HNF1A* mutation cases from the control group with an AUC of 0.90. Importantly, all single and derived antennary fucosylation traits provided a good discriminative power between pathogenic and benign mutation cases, as shown by AUCs ranging between 0.76 and 0.96. By applying these glycosylation traits, we were able to classify cases within the VUS group, in which one would have been categorized as carrying a damaging mutation type in the *HNF1A* gene, which was identified by significantly decreased fucosylation levels compared to the nonpathogenic and no mutation groups.

Significant overexpression of FUT6 has been reported in patients with non-alcoholic steatohepatitis (NASH), which is a progressive form of non-alcoholic fatty liver disease (NAFLD). Further, increased levels of fucosylated glycoprotein α-1 antitrypsin have been observed with pathological changes of NAFLD, such as fibrosis, pathological inflammation, and ballooning [47]. Due to the unavailability of specific markers of liver function in the clinical data of the collected sample cohort we were not able to determine the liver condition of individuals with diabetes enrolled in this study and a possible influence of liver dysfunction on antennary fucosylation levels. Although we have investigated a relationship between antennary fucosylation and CRP, which is also a predictor of NAFLD [48], a more in-depth understanding of inflammatory events that have a potential influence on the biomarker’s performance is needed. We acknowledge this aspect as a limitation of the current study, which should be investigated further.

In conclusion, glycan biomarkers have great potential in being applied to distinguish between healthy and diseased individuals. A large number of diseases including cancer and diabetes have been associated with changes in glycosylation profiles, which could be obtained through non- or minimally invasive analyses of body fluids and tissue samples [46, 49]. However, the expertise and high-end instrumentation that is required to detect these often subtle glycosylation changes are major obstacles in translation of novel glycan-based markers into clinical application. Moreover, the majority of diseases are the consequence of complex changes, for which combined diagnostic biomarkers are required [44]. Whereas, HNF1A-MODY is a disease for which a biomarker based solely on a glycosylation feature is sufficient to classify patients, as shown in the current and previous studies [17, 18].

Here, we have demonstrated the performance of a glycan-based biomarker for HNF1A-MODY across laboratories. This study confirms antennary fucosylation of N-glycans as a promising tool for patient stratification by enabling discrimination of cases with pathogenic mutations in the *HNF1A* gene compared to those with benign or variants of unknown significance. It is worth mentioning that the performance of antennary fucosylation as a biomarker for HNF1A-MODY could be influenced by inflammation events, as shown by the relationship between increased antennary fucosylation and CRP levels within (likely) damaging cases classified as outliers, therefore the significance of this observation should be further explored. Together, this is the first study which proves excellent robustness and reproducibility of the glycan biomarker for HNF1A-MODY tested on the same set of clinical samples across laboratories. The data presented here supports the development of a simpler, high-throughput assay for determining antennary fucosylation levels, which will facilitate future translation of this glycan biomarker into wider clinical practice.

## Supplementary Information

ESM 1 (DOCX 629 kb)

## Data Availability

The datasets generated for this study are available on request to the corresponding author.
