# Supplementary material for: Interlaboratory evaluation of plasma N-glycan antennary fucosylation as a clinical biomarker for HNF1A-MODY using liquid chromatography methods
Source: Glycoconj J. 2021 Mar 25;38(3):375–86. doi: 10.1007/s10719-021-09992-w (PMC8116301; doi:10.1007/s10719-021-09992-w)
Supplement: Supplementary file 1 — (DOCX 629 kb) [file 10719_2021_9992_MOESM1_ESM.docx]

**Supplementary Information**

**Interlaboratory evaluation of plasma N-glycan antennary fucosylation as a clinical biomarker for HNF1A-MODY using liquid chromatography methods**

Daniel Demus^1,2^*^*^*, Bas C. Jansen^1^, Richard A. Gardner^1^, Paulina A. Urbanowicz^1^, Haiyang Wu^3^, Tamara Štambuk^4,5^, Agata Juszczak*^6^*, Edita Pape Medvidović*^7^*, Nathalie Juge^3^, Olga Gornik^5^, Katharine R. Owen*^6^*^,8^, Daniel I. R. Spencer^1^*^*^*

1 Ludger Ltd, Culham Science Centre, Abingdon, Oxfordshire, England, United Kingdom

2 Center for Proteomics and Metabolomics, Leiden University Medical Center, Leiden, The Netherlands

3 Quadram Institute Bioscience, Norwich Research Park, United Kingdom

4 Genos Glycoscience Research Laboratory, Zagreb, Croatia

5 Faculty of Pharmacy and Biochemistry, University of Zagreb, Zagreb, Croatia

6 Oxford Centre for Diabetes, Endocrinology and Metabolism, University of Oxford, Oxford, Oxfordshire, England, United Kingdom

7 Vuk Vrhovac University Clinic for Diabetes, Endocrinology and Metabolic Diseases, Merkur University Hospital, Zagreb University School of Medicine, Zagreb, Croatia School of Medicine, Zagreb, Croatia

8 Oxford NIHR Biomedical Research Centre, Oxford Hospitals NHS Foundation Trust, Oxford, Oxfordshire, England, United Kingdom

* corresponding authors (daniel.demus@ludger.com, daniel.spencer@ludger.com)


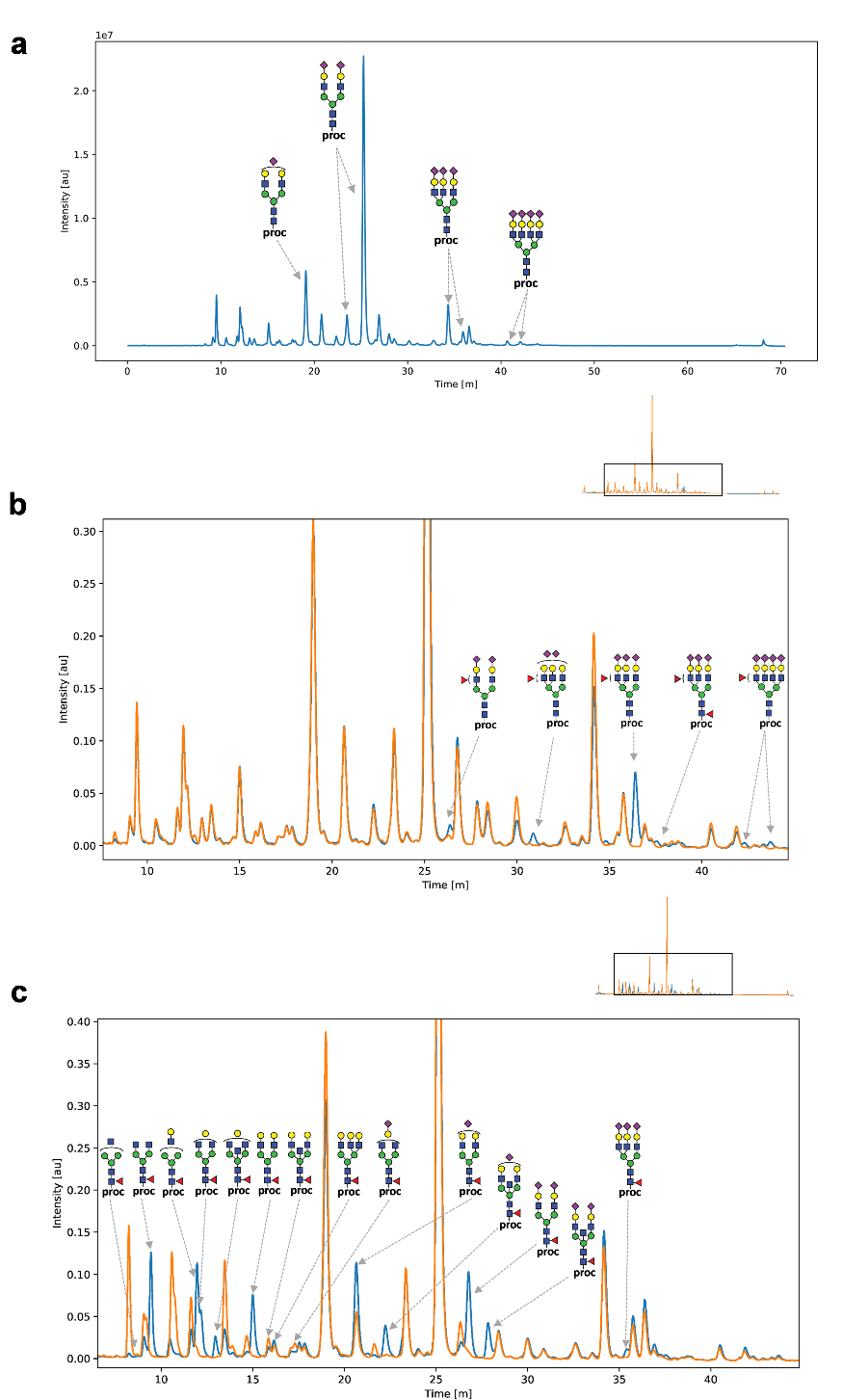


**Figure S1**. A representative fluorescence chromatogram of released procainamide labeled plasma N-glycans with main glycan peaks assigned (a) and fluorescence chromatograms obtained after exoglycosidase treatment with E1_10125 (b) and BKF (c). Displayed glycan structures indicate that the column groups glycans depending on their degree of sialylation. Chromatograms of enzymatically treated samples (orange) were overlaid with control samples (blue) and normalized to the highest peak. Zoomed areas show the glycan structure peak shifts. A multi-tool approach was applied to assign glycan structures to LC peaks. Structure assignment was based on both retention time and mass detection including manual examination of the MS/MS data. Fluorescence chromatograms obtained after treatment with two fucosidases with different substrate specificities were analysed for peak shifts that resulted in changes of peak relative intensity in comparison to controls (b-c). For graphical representations of glycan structures: blue square (*N*-acetylglucosamine), green circle (mannose), yellow circle (galactose), purple diamond (*N*-acetylneuraminic acid), red triangle (fucose).

**Figure S2. A fluorescence chromatogram of released procainamide labelled plasma N-glycans with numbered UHPLC peaks.** For a detailed list of UHPLC peaks with the most plausible glycan composition and possible glycan structure(s) see **Table S1**.

**Table S1. HPLC peak identification.** Identification was based on MS data inspection, literature knowledge [1] and exoglycosidase digestions. The table shows the most plausible glycan composition and possible glycan structure(s) of each HPLC peak together with theoretical and observed m/z values. Peak shifts that were observed as a result of exoglycosidase digestions were notified in this table as ✓ for complete digestion and ✓/- for incomplete digestion, which indicates another glycan structure under a peak.

| **Peak No.** | **Composition** | **Possible glycan** | **Possible structure** | **Theoretical m/z (charge)** | **Observed m/z (charge)** | **BKF digestion** | **E1_10125 fucosidase digestion** |
| --- | --- | --- | --- | --- | --- | --- | --- |
| **1** | **Hex3HexNAc4-Proc** | **A2** |  | **768.83**  **(2+)** | **768.73**  **(2+)** |  |  |
| **2** | **Hex4HexNAc3-Proc** | **FA1** |  | **748.32**  **(2+)** | **748.24**  **(2+)** |  |  |
| **3** | **Hex5HexNAc2-Proc** | **M5** |  | **727.81**  **(2+)** | **727.74**  **(2+)** |  |  |
|  | **Hex4HexNAc2-Proc** | **M4** |  | **646.78**  **(2+)** | **646.85**  **(2+)** |  |  |
| **4** | **Hex3HexNAc4dHex1-Proc** | **FA2** |  | **841.86**  **(2+)** | **841.79**  **(2+)** | **✓** |  |
| **5** | **Hex3HexNAc5dHex1-Proc** | **FA2B** |  | **943.40**  **(2+)** | **943.30**  **(2+)** | **✓** |  |
|  | **Hex4HexNAc4-Proc** | **A2G1** |  | **849.86**  **(2+)** | **849.77**  **(2+)** |  |  |
| **6** | **Hex5HexNAc3-Proc** | **M4A1G1** |  | **829.35**  **(2+)** | **829.26**  **(2+)** |  |  |
| **7** | **Hex6HexNAc2-Proc** | **M6 D1 OR D2**  **M6 D3** |  | **808.83**  **(2+)** | **808.76**  **(2+)** |  |  |
|  | **Hex4HexNAc5-Proc** | **A2BG1** |  | **951.40**  **(2+)** | **951.30**  **(2+)** |  |  |
| **8** | **Hex4HexNAc4dHex1-Proc** | **FA2G1** |  | **922.89**  **(2+)** | **922.80**  **(2+)** | **✓** |  |
|  | **Hex4HexNAc3dHex1-Proc** | **FA1G1** |  | **821.35**  **(2+)** | **821.38**  **(2+)** | **✓** |  |
|  | **Hex4HexNAc3NeuAc1-Proc** | **A1G1S1** |  | **893.87**  **(2+)** | **893.79**  **(2+)** |  |  |
| **9** | **Hex4HexNAc5dHex1-Proc** | **FA3G1**  **FA2BG1** |  | **683.28**  **(3+)**  **1024.43**  **(2+)** | **683.22**  **(3+)**  **1024.33**  **(2+)** | **✓** |  |
| **10** | **Hex5HexNAc4-Proc** | **A2G2** |  | **930.88**  **(2+)** | **930.80**  **(2+)** |  |  |
| **11** | **Hex6HexNAc3-Proc** | **M5A1G1** |  | **910.37**  **(2+)** | **910.40**  **(2+)** |  |  |
|  | **Hex4HexNAc3NeuAc1-Proc** | **A1G1S1** |  | **893.87**  **(2+)** | **893.79**  **(2+)** |  |  |
| **12** | **Hex7HexNAc2-Proc** | **M7 D3**  **M7D1** |  | **889.86**  **(2+)** | **889.76**  **(2+)** |  |  |
|  | **Hex5HexNAc5-Proc** | **A2BG2** |  | **688.62**  **(3+)** | **688.55**  **(3+)** |  |  |
| **13** | **Hex5HexNAc4dHex1-Proc** | **FA2G2** |  | **1003.91**  **(2+)**  **669.61**  **(3+)** | **1003.83**  **(2+)**  **669.54**  **(3+)** | **✓** |  |
| **14** | **Hex4HexNAc4NeuAc1-Proc** | **A2G1S1** |  | **995.41**  **(3+)**  **663.94**  **(3+)** | **995.32**  **(3+)**  **663.98**  **(3+)** |  |  |
|  | **Hex5HexNAc3NeuAc1-Proc** | **M4A1G1S1** |  | **974.89**  **(2+)** | **974.95**  **(2+)** |  |  |
| **15** | **Hex5HexNAc5dHex1-Proc** | **FA2BG2** |  | **737.30**  **(3+)** | **737.23**  **(3+)** | **✓** |  |
|  | **Hex6HexNAc5dHex1-Proc** | **FA3G3** |  | **791.32**  **(3+)** | **791.37**  **(3+)** |  |  |
| **16** | **Hex5HexNAc4NeuAc1-Proc** | **A2G2S1** |  | **717.95**  **(3+)** | **717.90**  **(3+)** |  |  |
|  | **Hex5HexNAc3NeuAc1-Proc** | **M4A1G1S1** |  | **974.89**  **(2+)** | **974.80**  **(2+)** |  |  |
| **17** | **Hex4HexNAc5NeuAc1-Proc** | **A2BG1S1**  **A3G1S1** |    | **731.63**  **(3+)** | **731.67**  **(3+)** | **✓/-** |  |
|  | **Hex4HexNAc4NeuAc1dHex1-Proc** | **FA2G1S1** |  | **1068.44**  **(2+)** | **1068.43**  **(2+)** |  |  |
| **18** | **Hex8HexNAc2-Proc** | **M8 D2,D3**  **M8 D1,D3** |  | **970.88**  **(2+)** | **970.80**  **(2+)** |  |  |
| **19** | **Hex5HexNAc4NeuAc1-Proc** | **A2G2S1** |  | **1076.43**  **(2+)** | **1076.33**  **(2+)** |  |  |
| **20** | **Hex6HexNAc3NeuAc1-Proc** | **M5A1G1S1** |  | **704.28**  **(3+)** | **704.32**  **(3+)** |  |  |
| **21** | **Hex5HexNAc4NeuAc1dHex1-Proc** | **FA2G2S1** |  | **766.64**  **(3+)** | **766.57**  **(3+)** | **✓** |  |
| **22** | **Hex5HexNAc5NeuAc1dHex1-Proc** | **FA2BG2S1**  **FA3G2S1** |    | **834.33**  **(3+)** | **834.30**  **(3+)** | **✓** |  |
| **23** | **Hex5HexNAc5NeuAc1-Proc** | **A2BG2S1**  **A3G2S1** |    | **785.65**  **(3+)** | **785.56**  **(3+)** |  |  |
|  | **Hex9HexNAc2-Proc** | **M9** |  | **1051.91**  **(2+)** | **1051.89**  **(2+)** |  |  |
| **24** | **Hex5HexNAc5NeuAc1dHex1-Proc** | **FA2BG2S1**  **FA3G2S1** |    | **834.33**  **(3+)** | **834.25**  **(3+)** | **✓** |  |
| **25** | **Hex5HexNAc4NeuAc2dHex1-Proc** | **FA2G2S2** |  | **863.67**  **(3+)** | **863.58**  **(3+)** | **✓** |  |
|  | **Hex5HexNAc4NeuAc2-Proc** | **A2G2S2** |  | **814.99**  **(3+)** | **814.91**  **(3+)** |  |  |
| **26** | **Not identified** |  |  | **-** | **-** |  |  |
| **27** | **Hex6HexNAc5NeuAc1-Proc** | **A3G3S1** |  | **839.67**  **(3+)** | **839.58**  **(3+)** |  |  |
| **28** | **Hex5HexNAc4NeuAc2dHex1-Proc** | **FA2G2S2** |  | **863.67**  **(3+)** | **863.59**  **(3+)** | **✓/-** |  |
|  | **Hex5HexNAc4NeuAc2-Proc** | **A2G2S2** |  | **814.99**  **(3+)** | **814.91**  **(3+)** |  |  |
| **29** | **Hex5HexNAc4NeuAc2dHex1-Proc** | **A2FG2S2** |  | **863.68**  **(3+)** | **863.60**  **(3+)** |  | **✓/-** |
|  | **Hex5HexNAc5NeuAc2-Proc** | **A2BG2S2** |  | **882.69**  **(3+)** | **882.68**  **(3+)** |  |  |
| **30** | **Hex5HexNAc4NeuAc2dHex1-Proc** | **FA2G2S2** |  | **863.67**  **(3+)** | **863.59**  **(3+)** | **✓** |  |
| **31** | **Hex5HexNAc5NeuAc2dHex1-Proc** | **FA2BG2S2**  **FA3G2S2** |  | **931.37**  **(3+)** | **931.28**  **(3+)** | **✓** |  |
| **32** | **Hex6HexNAc5NeuAc2-Proc** | **A3G3S2** |  | **936.70**  **(3+)** | **936.61**  **(3+)** |  |  |
| **33** | **Hex7HexNAc6NeuAc1-Proc** | **A4G4S1** |  | **961.38**  **(3+)** | **961.29**  **(3+)** |  |  |
| **34** | **Hex6HexNAc5NeuAc2-Proc** | **A3G3S2** |  | **936.70**  **(3+)** | **936.62**  **(3+)** |  |  |
| **35** | **Hex6HexNAc5NeuAc2dHex1-Proc** | **A3FG3S2** |  | **985.38**  **(3+)** | **985.29**  **(3+)** |  | **✓** |
| **36** | **Hex6HexNAc6NeuAc2-Proc** | **A4G3S2** |  | **1004.39**  **(3+)** | **1003.70**  **(3+)** |  |  |
| **37** | **Hex7HexNAc6NeuAc2-Proc** | **A4G4S2** |  | **794.06**  **(4+)** | **798.54**  **(4+)** |  |  |
| **38** | **Hex6HexNAc5NeuAc3-Proc** | **A3G3S3** |  | **1033.73**  **(3+)** | **1033.63**  **(3+)** |  |  |
| **39** | **Not identified** |  |  | **-** | **-** |  | **✓** |
| **40** | **Hex6HexNAc5NeuAc3dHex1-Proc** | **FA3G3S3** |  | **1082.42**  **(3+)**  **812.06**  **(4+)** | **1082.32**  **(3+)**  **812.34**  **(4+)** | **✓** |  |
| **41** | **Hex6HexNAc5NeuAc3-Proc** | **A3G3S3** |  | **1033.73**  **(3+)** | **1033.63**  **(3+)** |  |  |
| **42** | **Hex6HexNAc5NeuAc3dHex1-Proc** | **A3FG3S3** |  | **1082.42**  **(3+)**  **812.06**  **(4+)** | **1082.31**  **(3+)**  **812.25**  **(4+)** |  | **✓** |
| **43** | **Hex7HexNAc6NeuAc3-Proc** | **A4G4S3** |  | **866.83**  **(4+)** | **866.74**  **(4+)** |  |  |
| **44** | **Hex7HexNAc5NeuAc3dHex1-Proc** | **FA3FG3S3** |  | **1131.10**  **(3+)**  **848.58**  **(4+)** | **1130.97**  **(3+)**  **848.50**  **(4+)** |  | **✓** |
| **45** | **Hex7HexNAc6NeuAc3-Proc** | **A4G4S3** |  | **1155.44**  **(3+) 866.83**  **(4+)** | **1155.74**  **(3+)**  **866.75**  **(4+)** |  |  |
|  | **Not identified** |  |  | **-** | **-** |  | **✓/-** |
| **46** | **Hex7HexNAc6NeuAc4-Proc** | **A4G4S4** |  | **939.60**  **(4+)** | **939.58**  **(4+)** |  |  |
| **47** | **Hex7HexNAc6NeuAc4-Proc** | **A4G4S4** |  | **939.60**  **(4+)** | **939.76**  **(4+)** |  |  |
| **48** | **Hex7HexNAc6NeuAc4 dHex1-Proc** | **A4FG4S4** |  | **1301.16**  **(3+)**  **976.12**  **(4+)** | **1301.73**  **(3+)**  **975.99**  **(4+)** |  | **✓** |
| **49** | **Hex7HexNAc6NeuAc4 dHex2-Proc** | **A4FG4S4** |  | **976.12**  **(4+)** | **976.01**  **(4+)** |  | **✓** |

1. Saldova R, Asadi Shehni A, Haakensen VD, Steinfeld I, Hilliard M, Kifer I, et al. Association of N-glycosylation with breast carcinoma and systemic features using high-resolution quantitative UPLC. J Proteome Res. 2014;13: 2314–2327. doi:10.1021/pr401092y

**Table S2**. Technical variation of plasma N-glycan peaks used for statistical analysis with average relative intensities (RI), standard deviations (SD) and coefficient of variation (CV) values calculated based on standard plasma triplicates. The average relative intensities (RI), standard deviations (SD) and coefficient of variation (CV) were calculated based on pooled plasma triplicates for peaks used in statistical analysis within and between LC-MS runs.

|  | **p25** | **p28** | **p29** | **p32** | **p34** | **p35** | **p38** | **p40** | **p41** | **p42** | **p44** | **p46** | **p47** | **p48** | **p49** |
| --- | --- | --- | --- | --- | --- | --- | --- | --- | --- | --- | --- | --- | --- | --- | --- |
| **Plate 1** |  |  |  |  |  |  |  |  |  |  |  |  |  |  |  |
| RI | 0.0668 | 0.6474 | 0.0175 | 0.0257 | 0.0181 | 0.0085 | 0.1018 | 0.0072 | 0.0313 | 0.0422 | 0.0027 | 0.0122 | 0.0121 | 0.0027 | 0.0039 |
| SD | 0.0010 | 0.0097 | 0.0010 | 0.0019 | 0.0017 | 0.0004 | 0.0028 | 0.0007 | 0.0014 | 0.0020 | 0.0000 | 0.0008 | 0.0011 | 0.0002 | 0.0003 |
| CV (%) | 1% | 1% | 6% | 7% | 9% | 5% | 3% | 10% | 4% | 5% | 1% | 7% | 9% | 8% | 9% |
| **Plate 2** |  |  |  |  |  |  |  |  |  |  |  |  |  |  |  |
| RI | 0.0713 | 0.6625 | 0.0182 | 0.0269 | 0.0195 | 0.0090 | 0.0929 | 0.0062 | 0.0278 | 0.0378 | 0.0025 | 0.0102 | 0.0097 | 0.0022 | 0.0030 |
| SD | 0.0027 | 0.0094 | 0.0018 | 0.0036 | 0.0049 | 0.0018 | 0.0033 | 0.0004 | 0.0008 | 0.0007 | 0.0000 | 0.0003 | 0.0003 | 0.0003 | 0.0001 |
| CV (%) | 4% | 1% | 10% | 13% | 25% | 20% | 4% | 6% | 3% | 2% | 1% | 3% | 3% | 11% | 4% |
| **Plate 3** |  |  |  |  |  |  |  |  |  |  |  |  |  |  |  |
| RI | 0.0652 | 0.6514 | 0.0157 | 0.0276 | 0.0187 | 0.0093 | 0.0989 | 0.0062 | 0.0302 | 0.0433 | 0.0028 | 0.0120 | 0.0117 | 0.0025 | 0.0045 |
| SD | 0.0022 | 0.0094 | 0.0004 | 0.0019 | 0.0015 | 0.0007 | 0.0025 | 0.0001 | 0.0014 | 0.0025 | 0.0001 | 0.0009 | 0.0009 | 0.0003 | 0.0006 |
| CV (%) | 3% | 1% | 2% | 7% | 8% | 8% | 3% | 1% | 5% | 6% | 3% | 7% | 8% | 11% | 13% |
| **Plate 4** |  |  |  |  |  |  |  |  |  |  |  |  |  |  |  |
| RI | 0.0651 | 0.6405 | 0.0158 | 0.0222 | 0.0132 | 0.0069 | 0.0958 | 0.0063 | 0.0301 | 0.0413 | 0.0028 | 0.0118 | 0.0120 | 0.0028 | 0.0048 |
| SD | 0.0006 | 0.0063 | 0.0001 | 0.0002 | 0.0011 | 0.0003 | 0.0027 | 0.0006 | 0.0008 | 0.0015 | 0.0003 | 0.0003 | 0.0004 | 0.0004 | 0.0002 |
| CV (%) | 1% | 1% | 1% | 1% | 8% | 4% | 3% | 10% | 3% | 4% | 12% | 3% | 3% | 14% | 3% |
| **All plates** | |  |  |  |  |  |  |  |  |  |  |  |  |  |  |
| RI | 0.0671 | 0.6504 | 0.0168 | 0.0256 | 0.0174 | 0.0084 | 0.0974 | 0.0065 | 0.0299 | 0.0411 | 0.0027 | 0.0115 | 0.0114 | 0.0026 | 0.0040 |
| SD | 0.0029 | 0.0092 | 0.0012 | 0.0024 | 0.0028 | 0.0011 | 0.0038 | 0.0005 | 0.0015 | 0.0024 | 0.0001 | 0.0009 | 0.0011 | 0.0003 | 0.0008 |
| CV (%) | 4% | 1% | 7% | 9% | 16% | 13% | 4% | 7% | 5% | 6% | 5% | 8% | 10% | 10% | 19% |

**Table S3**. Performance of glycan traits in differentiating subjects with different *HNF1A* mutation status

| **Groups tested** | **Glycan trait** | **AUC** | **Cutoff** | **Sensitivity** | **Specificity** |
| --- | --- | --- | --- | --- | --- |
| (Likely) damaging *HNF1A* vs. no mutation | A2FG2S2 | 0.84 | 0.018 | 0.73 | 0.72 |
|  | A3FG3S2 | 0.94 | 0.101 | 0.93 | 0.87 |
|  | A3FG3S3 | 0.94 | 0.163 | 1.00 | 0.83 |
|  | FA3FG3S3 | 0.91 | 0.200 | 1.00 | 0.76 |
|  | A4FG4S4_I | 0.90 | 0.158 | 0.93 | 0.76 |
|  | A4FG4S4_II | 0.93 | 0.188 | 1.00 | 0.81 |
|  | Derived trait | 0.90 | 0.057 | 0.83 | 0.78 |
| (Likely) benign *HNF1A* vs. no mutation | A2FG2S2 | 0.54 | 0.033 | 0.86 | 0.25 |
|  | A3FG3S2 | 0.55 | 0.251 | 0.91 | 0.38 |
|  | A3FG3S3 | 0.58 | 0.381 | 0.90 | 0.38 |
|  | FA3FG3S3 | 0.57 | 0.481 | 0.94 | 0.38 |
|  | A4FG4S4_I | 0.63 | 0.264 | 0.89 | 0.38 |
|  | A4FG4S4_II | 0.60 | 0.421 | 0.93 | 0.38 |
|  | Derived trait | 0.55 | 0.107 | 0.82 | 0.50 |
| (Likely) damaging vs. (likely) benign *HNF1A* | A2FG2S2 | 0.85 | 0.023 | 1.00 | 0.63 |
|  | A3FG3S2 | 0.84 | 0.104 | 0.93 | 0.63 |
|  | A3FG3S3 | 0.92 | 0.168 | 1.00 | 0.63 |
|  | FA3FG3S3 | 0.76 | 0.224 | 1.00 | 0.63 |
|  | A4FG4S4_I | 0.96 | 0.165 | 1.00 | 0.75 |
|  | A4FG4S4_II | 0.94 | 0.196 | 1.00 | 0.63 |
|  | Derived trait | 0.83 | 0.069 | 1.00 | 0.63 |

**Table S4**. CRP levels and antennary fucosylation indexes calculated for damaging *HNF1A* and no *HNF1A* mutation cases included in the study (n = 15 and 267, respectively) and damaging *HNF1A* and no *HNF1A* mutation cases classified as data outliers (n = 3 and 21, respectively) excluded from the ROC analysis.

|  | **CRP level (mg/L)** | | | **Antennary fucosylation index** | | |
| --- | --- | --- | --- | --- | --- | --- |
|  | Study cases | Outliers | **p-value** | Study cases | Outliers | **p-value** |
| Damaging *HNF1A* mutation group | 0.72 (1.42) | 1.22 (0.46) | **0.075** | 0.033 (0.011) | 0.062 (0.004) | **0.0025** |
| No *HNF1A* mutation group | 3.80 (7.16) | 3.73 (3.90) | **0.364** | 0.076 (0.026) | 0.126 (0.042) | **6.3e-09** |

Values are presented as: mean (SD). The Wilcoxon-Mann-Whitney test was applied to compare the means; p ≤ 0.05 is considered statistically significant.

**Table S5**. The correlation analysis for glycan traits and CRP marker (n = 320).

| **Glycan trait** | **Spearman's correlation coefficient (r)** | **p-value** |
| --- | --- | --- |
| A2FG2S2 | -0.034 | 0.54 |
| A3FG3S2 | 0.14 | 0.011 |
| A3FG3S3 | 0.15 | 0.006 |
| FA3FG3S3 | 0.12 | 0.032 |
| A4FG4S4_I | 0.15 | 0.0079 |
| A4FG4S4_II | 0.18 | 0.0012 |
| Derived antennary fucosylation trait | 0.18 | 0.0015 |

p ≤ 0.05 is considered statistically significant
